# Association of atopic multimorbidity with childhood pet exposure and caesarean section delivery: a retrospective study from the Lifelines Cohort Study

**DOI:** 10.1093/skinhd/vzag070

**Published:** 2026-06-09

**Authors:** Rui Chen, Laura Loman, Douwe Postmus, Marie L A Schuttelaar

**Affiliations:** Department of Dermatology, University Medical Center Groningen, University of Groningen, Groningen, The Netherlands; Department of Dermatology, University Medical Center Groningen, University of Groningen, Groningen, The Netherlands; Department of Epidemiology, University Medical Center Groningen, University of Groningen, Groningen, The Netherlands; Department of Dermatology, University Medical Center Groningen, University of Groningen, Groningen, The Netherlands

## Abstract

**Background:**

The hygiene hypothesis suggests that reduced microbial exposure may contribute to the development of atopic diseases, whereas higher exposure may exacerbate symptoms and promote disease progression.

**Objectives:**

To investigate the association between multiple childhood environmental exposures and atopic multimorbidity, defined as atopic dermatitis (AD) accompanied by at least two other atopic comorbidities, including asthma, food allergy (FA) and allergic rhinitis (AR).

**Methods:**

Data on self-reported, physician-diagnosed lifetime AD were collected in 2020 through a digital questionnaire within the Lifelines Cohort Study, an ongoing population-based study including 167 729 residents of the northern Netherlands. Adults retrospectively reported childhood environmental exposures, including birthweight, gestational age, delivery mode, breastfeeding, tobacco smoke exposure (maternal or childhood), cat or dog ownership before the age of 16 years (ever owned, type and age at ownership) and living conditions. All exposure data, along with data on asthma, AR and FA, were collected between 2006 and 2013. Associations between environmental exposures and atopic multimorbidity (vs. no atopic disease) were assessed using logistic regression, adjusted for age, sex, socioeconomic status, parental history of asthma and sibling status.

**Results:**

Among 28 791 included participants, 27 939 (97.0%) reported no atopic diseases, while 852 (3.0%) reported atopic multimorbidity. Compared with participants without atopic diseases, caesarean section delivery was positively associated with atopic multimorbidity [odds ratio (OR) 2.55, 95% confidence interval (CI) 1.20–5.46], whereas pet ownership before the age of 16 years (OR 0.52, 95% CI 0.37–0.75), including ownership of dogs only (OR 0.59, 95% CI 0.37–0.94), cats only (OR 0.44, 95% CI 0.26–0.74) or both (OR 0.53, 95% CI 0.34–0.83), was negatively associated with atopic multimorbidity.

**Conclusions:**

Childhood pet ownership may be protective against atopic multimorbidity, whereas caesarean section delivery may be associated with a higher likelihood of atopic multimorbidity, providing insights into potential preventive strategies.

## Teaser

Based on data from the Lifelines cohort, 3.0% of 28 791 Dutch adults were found to have atopic multimorbidity. Childhood pet ownership, whether cats or dogs, was associated with a lower likelihood of atopic multimorbidity, whereas caesarean section delivery was associated with a higher likelihood. These findings provide insights into potential preventive strategies.

## Graphical Abstract

**Graphical Abstract:**
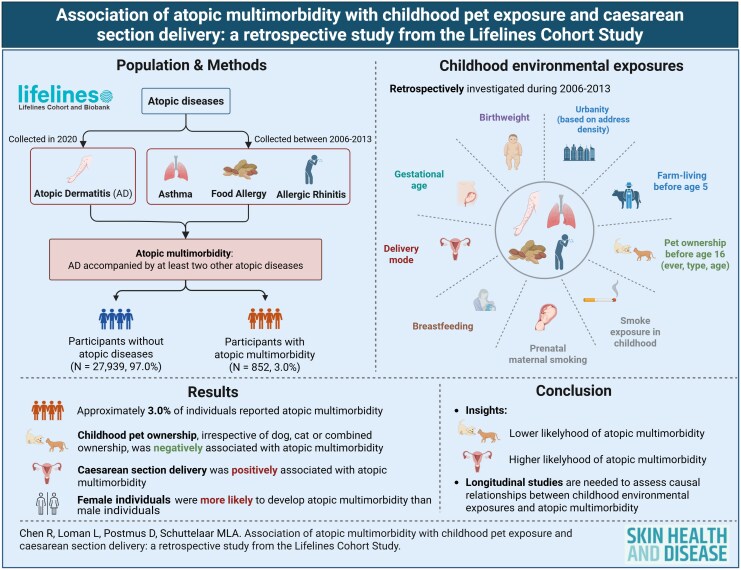

What is already known about this topic?

- Atopic multimorbidity is often seen in patients with atopic diseases.
- Childhood environmental exposures may either promote or protect against the development of atopic diseases.
- Evidence on the association between environmental exposures and atopic multimorbidity is limited and inconsistent.

What does this study add?

- In the Dutch general population, approximately 3.0% of individuals reported atopic multimorbidity.
- Childhood pet ownership, including cats or dogs, compared with no pet ownership, was negatively associated with atopic ­multimorbidity, whereas caesarean section delivery, compared with vaginal delivery, was positively associated with atopic multimorbidity.

Atopic diseases, including atopic dermatitis (AD), asthma, allergic rhinitis (AR) and food allergy (FA), are common chronic health conditions that often coexist within individuals, a phenomenon known as atopic multimorbidity.^1,2^ The prevalence of atopic diseases has been increasing rapidly worldwide.^3^ This trend is thought to be influenced by changes in environmental and lifestyle factors such as improved hygiene, urbanization, reduced microbial diversity and altered early-life exposures.^4,5^ Moreover, substantial geographical variation in prevalence further supports the importance of environmental determinants in the development of atopic diseases.^6,7^

Environmental exposures cover a wide range of factors. Previously, factors such as maternal and *in utero* exposures, delivery mode, birthweight, gestational age, tobacco smoke exposure, breastfeeding, pet ownership, presence of siblings, rural or urban living, outdoor and indoor air pollutants, climate, microbial exposure, and the use of probiotics and prebiotics have been recognized to significantly influence the phenotypes of atopic diseases and may contribute to their onset and/or progression.^8,9^ Moreover, the timing of exposure, especially during early life, is considered important in the development and progression of atopic diseases.^10–12^ Although numerous studies have examined childhood environmental exposures in relation to atopic diseases, evidence regarding whether certain environmental factors exacerbate or protect against the development of these diseases remain inconclusive.

For instance, early-life exposure to pets is often considered a protective factor against atopic diseases, aligning with the ‘hygiene hypothesis’, which suggests that greater microbial exposure in early childhood may reduce susceptibility to atopic diseases.^13^ However, a meta-analysis of European birth cohorts reported no significant overall association between early-life pet ownership and asthma or pet-specific allergic sensitization in school-aged children.^14^ Similarly, despite links between urbanization and atopic risk, meta-analyses do not consistently show a direct association with air pollution.^15,16^

Understanding the relationship between environmental exposures and atopic multimorbidity is crucial for developing effective prevention strategies and interventions. While prior research has largely focused on individual atopic diseases, primarily in paediatric populations, studies investigating the impact of childhood environmental exposures on atopic multimorbidity remain limited and have yielded conflicting conclusions.^17,18^ Therefore, this study aims to investigate the associations between childhood environmental exposures and the likelihood of developing atopic multimorbidity in the Dutch general population.

## Materials and methods

### Study population

The current study was conducted within the Lifelines cohort study, a multidisciplinary prospective population-based cohort study examining the health and health-related behaviours of 167 729 persons living in the north of the Netherlands in a unique three-generation design.^19^

Data on self-reported, physician-diagnosed lifetime AD were collected through a digital add-on skin questionnaire (SKIQ), which was sent to all adult Lifelines participants (*n* = 135 950) between February and May 2020, achieving a response rate of 42.4% (*n* = 57 643).^20^ Information on lifetime asthma, FA and AR was collected during the Lifelines baseline assessment, conducted between 2006 and 2013.

### Outcomes

Atopic diseases often interact and tend to develop sequentially, typically beginning with AD in early life.^1^ To identify individuals with a more clinically significant and complex disease burden, and to reduce misclassification due to isolated or transient comorbidities, atopic multimorbidity was defined as AD accompanied by at least two of the following atopic diseases: asthma, FA or AR.

Details of the diagnostic criteria for AD, asthma, FA and AR have been described in a previous Lifelines study.^21^ In brief, self-administered questionnaires assessing lifetime prevalence of the four atopic diseases were used. Participants with AD were identified as those reporting physician-diagnosed AD in their lifetime. Asthma was defined as either self-reported physician-diagnosed lifetime asthma or the presence of at least two symptoms (wheeze, shortness of breath at rest or nocturnal shortness of breath), based on the European Community Respiratory Health Survey questionnaire,^22^ and current use of asthma medication, identified according to the Anatomical Therapeutic Chemical code.^23^ FA was defined as self-reporting at least one food allergen and at least one symptom consistent with immediate allergic reactions to food along with other characteristics of FA consistent with immediate allergic reactions to food.^24^ AR was identified based on self-reported nasal allergies (including hay fever).

Based on the identification of AD, asthma, FA and AR, the current study population included individuals with either no atopic diseases or with atopic multimorbidity. Participants who did not fit into either category were excluded from the analysis, including those with AD only, AD plus one additional atopic comorbidity, and those without AD but reporting any of the other three atopic diseases.

### Childhood environmental exposures

All environmental exposures included in the current study are listed in Table S1 (see Supporting Information). The following exposures were included: birthweight, gestational age, mode of delivery, ever being breastfed, tobacco smoke exposure (including prenatal maternal smoking and childhood exposure), pet exposure before the age of 16 years (cats and/or dogs only), including ever ownership (yes/no), type of pet (cat, dog or both) and age at ownership (<1 year or 1–16 years), as well as living conditions (including farm/nonfarm residence and urban/rural residence). Urbanity level (urban/rural) was classified based on addresses density, with urban areas defined as having ≥1000 addresses per km^2^ and rural areas as having <1000 addresses per km^2^.^25^ All other childhood environmental exposures were retrospectively reported by adult participants between 2016 and 2013.

### Potential confounders

Age was categorized into three groups based on the first and third quartiles of the age distribution: 25–49 years (below the first quartile; 23.4%), 50–65 years (between the first and third quartiles; 50.8%) and ≥66 years (above the third quartile; 25.8%).

Socioeconomic status (SES) was assessed using neighbourhood socioeconomic status (NSES) scores, which were calculated based on residents’ average educational level, income and employment prospectives, and ranged from −8 to +3.^26^ NSES scores were categorized into three levels: low (NSES < −1), middle (NSES −1 to +1) and high (NSES > + 1).^27^

Self-reported parental history of asthma (≥1 parent vs. none) and sibling status (≥1 sibling vs. none) were also included as potential confounders.

### Data analysis

Environmental exposures were described as mean (SD) for continuous variables with a normal distribution, and as numbers and proportions for categorical variables. For subgroup comparisons, independent samples *t*-tests and X^2^ tests were used for continuous variables with normal distribution and for categorical variables, respectively. Logistic regression models were used to explore the associations between atopic multimorbidity and environmental exposures. For each environmental exposure, three models were constructed: (i) an unadjusted model; (ii) Model 1, adjusted for age and sex; and (iii) Model 2, additionally adjusted for NSES, parental history of asthma and sibling status. Results are shown as odds ratios (ORs) with 95% confidence intervals (CIs).

To assess the robustness of the findings, two sensitivity analyses were performed. Firstly, given that childhood exposures were retrospectively reported, recall bias may be present and could vary by age. Exposure × age group interaction terms were therefore included to assess potential effect heterogeneity. Interaction terms were retained in Model 2 only when statistically significant. Secondly, nonresponder analyses were conducted to compare the study population with all remaining adult participants in the Lifelines cohort who were not included in the study. Depending on the variable type, effect sizes were calculated to quantify the magnitude of group differences: Cohen’s *d* for continuous variables, Cohen’s *h* for binary variables and Cramer’s *V* for multilevel cat­egorical variables. The interpretation of effect sizes followed ­conventional thresholds proposed by Cohen, where values of approximately 0.2, 0.5 and 0.8 for Cohen’s *d* (or *h*), and 0.1, 0.3 and 0.5 for Cramer’s *V*, indicate small, medium and large effects, respectively.^28^

Data analysis and visualization were performed using SPSS Statistics (version 28.0; IBM, Armonk, NY, USA) and R (version 4.4.0). A two-tailed *P*-value < 0.05 was considered statistically significant.

## Results

**Figure 1:**
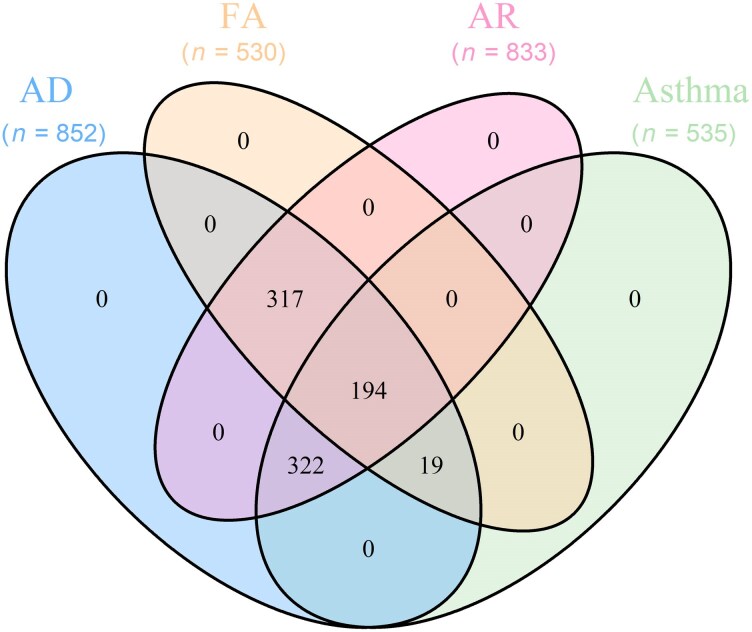
Venn diagram of overlapping atopic diseases among participants with atopic multimorbidity (*n* = 852). AD, atopic dermatitis; AR, allergic rhinitis; FA, food allergy.

Overall, 44 559 participants had complete information on all 4 atopic diseases (Figure S1; see Supporting Information). After excluding participants with AD only (*n* = 1830), those with AD and one additional atopic comorbidity (*n* = 1289) and those without AD but with any of the other three atopic diseases (*N* = 12 649), a total of 28 791 participants were included in the analysis as the study population. Of these, 27 939 participants (97.0%) had no atopic diseases, while 852 participants (3.0%) had atopic multimorbidity (Table 1). Among the 852 participants with atopic multimorbidity, 322 (37.8%) had AD, asthma and AR; 317 (37.2%) had AD, FA and AR; 19 (2.2%) had AD, FA and asthma; and the remaining 194 (22.8%) had all 4 atopic diseases (Figure 1).

**Table 1:** Description of study population

	Study population (*n* = 28 791)	No atopic diseases (*n* = 27 939; 97.0%)	Atopic multimorbidity (*n* = 852; 3.0%)	*P*-value
Age (years), mean (SD)	57.28 (11.89)	57.46 (11.86)	51.13 (11.41)	**<0**.**001**
Sex				**<0**.**001**
Male	11 918 (41.4)	11 722 (42.0)	196 (23.0)	
Female	16 873 (58.6)	16 217 (58.0)	656 (77.0)	
Neighbourhood socioeconomic status				**0**.**04**
Low	8039 (32.2)	7763 (32.1)	276 (36.5)	
Intermediate	15 624 (62.6)	15 180 (62.7)	444 (58.7)	
High	1314 (5.3)	1278 (5.3)	36 (4.8)	
Missing	3814	3718	96	
Parental asthma status				**<0**.**001**
No	1359 (46.8)	1326 (48.4)	33 (20.0)	
Yes	1543 (53.2)	1411 (51.6)	132 (80.0)	
Missing	25 889	25 202	687	
Sibling status				>0.90
No	53 (0.2)	52 (0.2)	1 (0.1)	
Yes	25 867 (99.8)	25 118 (99.8)	749 (99.9)	
Missing	2871	2769	102	
Birthweight (kg)				0.76
Low, <2.5	1443 (7.7)	1389 (7.7)^a^	54 (8.4)^a^	
Normal, 2.5–4	15 184 (80.8)	14 670 (80.8)^a^	514 (79.8)^a^	
High, >4	2168 (11.5)	2092 (11.5)^a^	76 (11.8)^a^	
Missing	9996	9788	208	
Gestational age (weeks)				**<0**.**001**
Preterm, <37	919 (4.6)	884 (4.6)^a^	35 (5.1)^a^	
Early term, 37–38	1580 (7.9)	1520 (7.9)^a^	60 (8.8)^a^	
Full term, 39–40	14 130 (70.9)	13 697 (71.2)^a^	433 (63.3)^b^	
Late/post-term, ≥41	3290 (16.5)	3134 (16.3)^a^	156 (22.8)^b^	
Missing	8872	8704	168	
Delivery mode				**0**.**004**
Vaginal delivery	27 210 (98.0)	26 406 (98.1)	806 (96.6)	
Caesarean section	553 (2.0)	525 (1.9)	28 (3.4)	
Missing	1028	1010	18	
Breastfeeding ever				0.54
No	5501 (34.2)	5292 (34.2)	209 (35.4)	
Yes	10 561 (65.8)	10 180 (65.8)	381 (64.6)	
Missing	12 729	12 467	262	
Prenatal maternal smoking				**<0**.**001**
No	21 515 (86.6)	20 921 (86.8)	594 (80.4)	
Yes	3323 (13.4)	3178 (13.2)	145 (19.6)	
Missing	3953	3840	113	
Smoke exposure in childhood				**<0**.**001**
No	5888 (23.7)	5667 (23.5)	221 (29.9)	
Yes	18 950 (76.3)	18 432 (76.5)	518 (70.1)	
Missing	3953	3840	113	
Pet ownership ever				**0**.**004**
No	8274 (28.8)	7991 (28.6)	283 (33.2)	
Yes	20 495 (71.2)	19 926 (71.4)	569 (66.8)	
Missing	22	22	0	
Type of pet				0.08
Dog only	6343 (30.9)	6144 (30.8)^a^	199 (35.0)^b^	
Cat only	5742 (28.0)	5599 (28.1)^a^	143 (25.1)^a^	
Both dog and cat	8410 (41.0)	8183 (41.1)^a^	227 (39.9)^a^	
Age of having pet				0.07
During the first year	9785 (47.7)	9492 (47.6)	293 (51.5)	
1–16 years old	10 710 (52.3)	10 434 (52.4)	276 (48.5)	
Farm living before 5 years				**<0**.**001**
Nonfarm	23 451 (83.6)	22 690 (83.3)	761 (92.1)	
Farm	4608 (16.4)	4543 (16.7)	65 (7.9)	
Missing	732	706	26	
Urbanity, addresses per km^2^				**<0**.**001**
Urban (≥1000)	8766 (30.4)	8445 (30.2)	321 (37.7)	
Rural (<1000)	20 025 (69.6)	19 494 (69.8)	531 (62.3)	

*Data are presented as *n* (%) unless otherwise stated. P*-value indicates the difference between participants with atopic multimorbidity and those without any atopic diseases; *P* < 0.05 was considered statistically significant and is shown in bold. ^a, b^Values with different superscript letters indicate that the proportions differ significantly between participants with atopic multimorbidity and those without any atopic diseases at the 0.05 level; values sharing the same superscript letter do not differ significantly.

Compared with participants without atopic diseases, those with atopic multimorbidity were significantly younger [mean (SD) 51.13 (11.41) years vs. 57.46 (11.86)], and a higher proportion were women [77.0% (*n* = 656/852) vs. 58.0% (*n* = 16 217/27 939)] (all *P* < 0.001; Table 1).

The impact of childhood environmental exposures on atopic multimorbidity was further investigated using logistic regression analysis (Table 2). After adjustment for potential confounders (Model 2), compared with participants without any atopic diseases, delivery by caesarean section was associated with higher odds of atopic multimorbidity (OR 2.55, 95% CI 1.20–5.46). In contrast, ever owning a pet before the age of 16 years was associated with lower odds of atopic multimorbidity (OR 0.52, 95% CI 0.37–0.75), regardless of whether participants owned dogs only (OR 0.59, 95% CI 0.37–0.94), cats only (OR 0.44, 95% CI 0.26–0.74), or both dogs and cats (OR 0.53, 95% CI 0.34–0.83) (all *P* < 0.05). Farm living before the age of 5 years and residence in a rural area were associated with lower odds of atopic multimorbidity in Model 1; however, these associations were no longer statistically significant after further adjustment for NSES, parental asthma status and sibling status (Model 2). Furthermore, female sex was significantly associated with higher odds of atopic multimorbidity compared with male sex (results not shown).

**Table 2:** Logistic regression analysis of childhood environmental exposures and atopic multimorbidity

Variable	Variable levels	Crude model		Model 1		Model 2	
		OR (95% CI)	*P*-value	OR (95% CI)	*P*-value	OR (95% CI)	*P-*value
Age		**0.96 (0.95–0.96)**	**<0**.**001**				
Sex	Female (vs. male)	**2.42 (2.06–2.85)**	**<0**.**001**				
NSES	Intermediate (vs. low)	**0.82 (0.71–0.96)**	**0**.**01**				
	High (vs. low)	0.79 (0.55–1.11)	0.20				
Parental history of asthma	Yes (vs. none)	**3.76 (2.58–5.63)**	**<0**.**001**				
Sibling status	Yes (vs. none)	1.55 (0.34–27.5)	0.66				
Birthweight	Low (vs. normal)	1.11 (0.83–1.48)	0.48	1.15 (0.86–1.54)	0.33	0.93 (0.48–1.82)	0.83
	High (vs. normal)	1.04 (0.81–1.32)	0.77	1.19 (0.93–1.52)	0.17	1.02 (0.54–1.91)	0.96
Gestational age	Preterm (vs. full term)	1.25 (0.88–1.78)	0.21	1.11 (0.78–1.58)	0.56	1.47 (0.66–3.31)	0.35
	Early term (vs. full term)	1.25 (0.95–1.64)	0.11	0.98 (0.74–1.29)	0.87	0.72 (0.34–1.52)	0.39
	Late/post-term (vs. full term)	1.57 (1.31–1.90)	**<0**.**001**	1.20 (0.99–1.46)	0.06	1.50 (0.95–2.37)	0.08
Delivery mode	Caesarean section (vs. vaginal delivery)	1.75 (1.19–2.57)	**0**.**005**	1.18 (0.80–1.75)	0.41	**2.55 (1.20–5.46)**	**0**.**02**
Breastfeeding ever	Yes (vs. no)	0.95 (0.80–1.13)	0.54	1.02 (0.86–1.22)	0.80	1.14 (0.73–1.78)	0.56
Prenatal maternal smoking	Yes (vs. none)	1.61 (1.34–1.93)	**<0**.**001**	1.16 (0.96–1.40)	0.13	1.18 (0.72–1.94)	0.51
Smoke exposure in childhood	Yes (vs. none)	0.72 (0.61–0.85)	**<0**.**001**	0.96 (0.81–1.13)	0.63	0.95 (0.63–1.44)	0.81
Pet ownership ever	Yes (vs. none)	**0.81 (0.70–0.93)**	**0**.**004**	**0.76 (0.65–0.88)**	**<0**.**001**	**0.52 (0.37–0.75)**	**<0**.**001**
Type of pet	Dog only (vs. no pet)	0.91 (0.76–1.10)	0.34	0.88 (0.73–1.06)	0.19	**0.59 (0.37–0.94)**	**0**.**03**
	Cat only (vs. no pet)	**0.72 (0.59–0.88)**	**0**.**002**	**0.66 (0.54–0.81)**	**<0**.**001**	**0.44 (0.26–0.74)**	**0**.**002**
	Both dog and cat (vs. no pet)	**0.78 (0.66–0.94)**	**0**.**007**	**0.73 (0.61–0.87)**	**<0**.**001**	**0.53 (0.34–0.83)**	**0**.**006**
Age of having pet	1–16 years (vs. <1 years)	0.86 (0.73–1.01)	0.07	1.01 (0.85–1.19)	0.94	0.82 (0.51–1.30)	0.39
Farm living before 5 years	Yes (vs. no)	**0.43 (0.33–0.55)**	**<0**.**001**	**0.49 (0.38–0.64)**	**<0**.**001**	0.72 (0.40–1.28)	0.27
Urbanity living	Rural (vs. urban)	**0.72 (0.62–0.83)**	**<0**.**001**	**0.75 (0.65–0.87)**	**<0**.**001**	0.72 (0.50–1.04)	0.08

Model 1: adjusted for age and sex; Model 2: adjusted for age, sex, neighbourhood socioeconomic status (NSES), parental history of asthma and sibling status. *P* < 0.05 was considered statistically significant and is shown in bold. CI, confidence interval; OR, odds ratio.

Sensitivity analysis indicated that neither the main effect of age group nor the interaction between age group and childhood exposures was statistically significant. The ORs and 95% CIs for each exposure by age group are shown in Figure S2 (see Supporting Information). Moreover, nonresponder analysis indicated that the study population was significantly older and included a higher proportion of women (Table S2; see Supporting Information). Although environmental exposures during childhood differed between groups (all *P* < 0.05), all effect sizes were below 0.2, suggesting that these differences were small and probably driven by the large sample size rather than by meaningful selection bias.

## Discussion

In total, 3.0% of the study population reported having atopic multimorbidity, defined as self-reported physician-diagnosed AD combined with at least two other atopic diseases. Caesarean section delivery was associated with higher odds of atopic multi­morbidity, whereas pet ownership before the age of 16 years, regardless of whether individuals owned cats, dogs or both, was associated with lower odds. In addition, women had higher odds of atopic multimorbidity compared with men.

Population-based cohort studies suggest that exposure to cats and dogs in early childhood may be associated with a reduced risk of developing multiple atopic conditions, including AD, asthma and AR, supporting a potential role of pet-related microbial exposures in immune maturation.^29,30^ A prospective population-based Finnish birth cohort study showed that exposure to a household dog during the first year of life was associated with a 23–40% lower risk of developing asthma, AR and AD development by the age of 5 years, and household cat exposure may protect against AD later in childhood.^30^ However, the evidence remains inconsistent. A pooled analysis from 11 European birth cohorts found no significant association between early-life exposure to furry or feathered pets and subsequent development of asthma or AR.^31^ In line with these epidemiological findings, genetic studies further highlight the importance of gene–environment interactions in atopic disease development. A large European study involving approximately 280 000 individuals investigated interactions between 24 known eczema risk variants and 18 environmental factors and found that early-life dog exposure may modify the genetic effect of a specific loci, thereby supporting a protective effect of environmental exposures.^32^ In contrast, a Dutch birth cohort study suggested that early-life exposure to cats may enhance the effect of filaggrin gene mutations on the development of eczema and sensitization.^33^ Collectively, these findings highlight the importance of gene–environment interactions in development of atopic disease.

Beyond the overall effect of pet exposure and pet type, the timing of exposure also plays an important role. A prospective Danish cohort study demonstrated that exposure to cats or dogs, either limited to early childhood or extending from pregnancy through early childhood, was associated with a slightly reduced risk of developing asthma and AR.^29^ Consistently, a Norwegian population-based birth cohort reported that early-life pet exposure was protective against the development of AD, asthma and AR during childhood.^34^ However, evidence from other large-scale studies suggests that these associations may be context-dependent. Findings from the European Community Respiratory Health Survey, conducted across more than 30 European regions, showed that childhood cat ownership was linked to asthma exclusively in individuals with atopy, with stronger associations observed in areas with relatively low community-level cat exposure.^35,36^ In contrast, a recent meta-analysis including more than 77 000 children from the EU Child Cohort Network found no independent association between early-life exposure to cats or dogs and school-age asthma, with neither the timing nor the intensity of exposure clearly influencing the results.^14^ Similarly, a retrospective cohort study from China investigating childhood doctor-­diagnosed asthma in relation to indoor environmental exposure across four critical time windows (1 year before pregnancy, during pregnancy, the first year after birth and the past year) reported no significant associations between asthma and ownership of furry pets (dogs or cats) during any of these periods.^37^

Taken together, these findings suggest that the relationship between childhood pet ownership and later atopic outcomes is shaped by a complex interplay between genetic susceptibility, allergic sensitization, timing of exposure and the broader environmental allergen context.^35^ Moreover, beyond the potential impact of pet exposure itself, heterogeneity across studies may be partly attributable to differences in questionnaire-based ascertainment of pet exposure, including variations in type (direct or indirect), timing, duration and intensity.^38^

In addition, perinatal factors such as delivery mode have also been implicated in the development of atopic diseases. Caesarean section delivery has been found to be associated with an increased risk of atopic diseases, including AD, asthma, AR and FA, compared with vaginal delivery.^39–42^ A comprehensive systematic review and meta-analysis including over 100 observational studies reported that children born by caesarean section had significantly higher risks of asthma, AR, AD, FA and allergic sensitization, with ORs ranging from approximately 1.08 to 1.35.^42^ Similar findings have been observed in a nationwide Swedish register-based study, where caesarean section was associated with a 12% increased risk of early childhood AD; however, after adjustment for sibling effects the association remained significantly only in children younger than 1 year.^43^

In the current study, farm living was inversely associated with atopic multimorbidity in age- and sex-adjusted models, but not after additional adjustment for SES, parental history of asthma and sibling status. This attenuation suggests that the apparent protective effect of farm living may be partly explained by correlated familial and socioeconomic factors rather than farm exposure itself. Larger family size and lower parental atopic predisposition, both more common in farm households,^44^ are associated with reduced atopic risk and may account for part of the observed association.^45,46^ Data from the Copenhagen Prospective Study on Asthma in Childhood (COPSAC) cohort, a prospective mother–child cohort in Copenhagen comprising 700 children, showed that infants raised in urban environments generally had a significantly higher prevalence of asthma, eczema, AR and aeroallergen sensitivity at the age of 6 years than those living in rural areas.^44^ However, after adjusting for lifestyle factors such as SES, pet ownership, the presence of older siblings, breastfeeding duration and passive smoking exposure, a positive association only remained for asthma and aeroallergen sensitization in urban infants,^44^ ­highlighting that the protective effects of farm exposure may be disease-specific rather than extending to atopic multimorbidity.^47^

Additionally, previous clinical and epidemiology studies have consistently reported a higher prevalence of atopic conditions in female individuals than in male individuals, which aligns with the current findings.^48,49^ However, this sex difference varies across age groups: atopic diseases are more common in boys during childhood, but this pattern reverses after puberty.^50,51^ Increasing evidence suggests that biologic, immunological and hormonal factors may contribute to this difference, including the effects of female sex hormones on immune memory and airway inflammation.^52,53^ Moreover, hormonal fluctuations throughout the menstrual cycle, pregnancy and menopause, can influence immune function, further increasing the susceptibility to allergic diseases among female individuals.^52^

The main strengths of this study include the comprehensive assessment of childhood environmental exposures and the focus on atopic multimorbidity, for which evidence remains limited. Regarding the limitations, firstly, the cross-sectional design limits the ability to infer causality. Secondly, childhood exposures were retrospectively reported, introducing potential recall bias. However, no significant interaction with age group was observed, suggesting limited age-related recall bias. Thirdly, as the study included only residents of the northern Netherlands, generalizability may be limited. The region’s moderate urban–rural (approximately 37.0% vs. 63.1%) gradient and relatively low pet ownership in the Netherlands may have limited the exposure variability, potentially attenuating the observed associations.^54,55^ In addition, selection bias related to the 42.4% response rate to AD-related questions in the SKIQ cannot be excluded,^20^ although nonresponder analyses showed small absolute differences and comparable exposure distribution. Lastly, several potential confounders, such as parental history of AD, AR and FA, as well as daycare attendance, were not included and may have influenced the findings. In particular, pet avoidance among parents who were atopic could have led to an overestimation of the observed negative associations, and residual confounding by familial atopic predisposition and early-life factors such as daycare attendance cannot be excluded.

In conclusion, individuals exposed to pets before the age of 16 years, whether cats only, dogs only or both, were less likely to develop atopic multimorbidity, whereas those delivered by caesarean sections had a higher likelihood. These findings support further exploration of preventive approaches that promote controlled microbial exposure, such as interaction with pets, during early childhood and primary school environments. Furthermore, as environmental exposure may differentially influence atopic multimorbidity and individual atopic diseases, longitudinal studies are needed to further investigate causal relationships and underlying mechanisms.

## Supplementary Material

vzag070_Supplementary_Data

## Data Availability

The data used in this study are available on request from the Lifelines Biobank [Lifelines, de biobank van Noord-Nederland | Lifelines (https://www.lifelines.nl/)]. The data are not publicly available due to privacy and ethical reasons.

## References

[1] Mrkić Kobal I, Plavec D, Vlašić Lončarić Ž et al Atopic march or atopic multimorbidity-overview of current research. Medicina (Kaunas) 2023; 60:21. 38256282 10.3390/medicina60010021 PMC10819021

[2] Diaz-Cabrera NM, Sánchez-Borges MA, Ledford DK. Atopy: a collection of comorbid conditions. J Allergy Clin Immunol Pract 2021; 9:3862–6. 34509674 10.1016/j.jaip.2021.09.002

[3] Asher MI, Montefort S, Björkstén B et al Worldwide time trends in the prevalence of symptoms of asthma, allergic rhinoconjunctivitis, and eczema in childhood: ISAAC Phases One and Three repeat multicountry cross-sectional surveys. Lancet 2006; 368:733–43. 16935684 10.1016/S0140-6736(06)69283-0

[4] Augustine T, Kumar M, Al Khodor S et al Microbial dysbiosis tunes the immune response towards allergic disease outcomes. Clin Rev Allergy Immunol 2023; 65:43–71. 35648372 10.1007/s12016-022-08939-9 PMC10326151

[5] Schoos AM . Atopic diseases – diagnostics, mechanisms, and exposures. Pediatr Allergy Immunol 2024; 35:e14198. 39016386 10.1111/pai.14198

[6] Dierick BJH, van der Molen T, Flokstra-de Blok BMJ et al Burden and socioeconomics of asthma, allergic rhinitis, atopic dermatitis and food allergy. Expert Rev Pharmacoecon Outcomes Res 2020; 20:437–53. 32902346 10.1080/14737167.2020.1819793

[7] Celebi Sözener Z, Cevhertas L, Nadeau K et al Environmental factors in epithelial barrier dysfunction. J Allergy Clin Immunol 2020; 145:1517–28. 32507229 10.1016/j.jaci.2020.04.024

[8] Burbank AJ, Sood AK, Kesic MJ et al Environmental determinants of allergy and asthma in early life. J Allergy Clin Immunol 2017; 140:1–12. 28673399 10.1016/j.jaci.2017.05.010 PMC5675123

[9] Kantor R, Silverberg JI. Environmental risk factors and their role in the management of atopic dermatitis. Expert Rev Clin Immunol 2017; 13:15–26. 27417220 10.1080/1744666X.2016.1212660 PMC5216178

[10] Murrison LB, Brandt EB, Myers JB et al Environmental exposures and mechanisms in allergy and asthma development. J Clin Invest 2019; 129:1504–15. 30741719 10.1172/JCI124612 PMC6436881

[11] Lu C, Li Q, Qiao Z et al Early and late onset childhood eczema: role of preconceptional, pre-natal and post-natal environmental exposures. Build Environ 2024; 258:111626.

[12] Lu C, Liu Z, Liao H et al Effects of early life exposure to home environmental factors on childhood allergic rhinitis: modifications by outdoor air pollution and temperature. Ecotoxicol Environ Saf 2022; 244:114076. 36113271 10.1016/j.ecoenv.2022.114076

[13] Strachan DP . Family size, infection and atopy: the first decade of the “hygiene hypothesis”. Thorax 2000; 55:S2–10. 10943631 10.1136/thorax.55.suppl_1.s2 PMC1765943

[14] Pinot de Moira A, Strandberg-Larsen K, Bishop T et al Associations of early-life pet ownership with asthma and allergic sensitization: a meta-analysis of more than 77,000 children from the EU Child Cohort Network. J Allergy Clin Immunol 2022; 150:82–92. 35150722 10.1016/j.jaci.2022.01.023

[15] Jackson CM, Kaplan AN, Järvinen KM. Environmental exposures may hold the key; impact of air pollution, greenness, and rural/farm lifestyle on allergic outcomes. Curr Allergy Asthma Rep 2023; 23:77–91. 36609951 10.1007/s11882-022-01061-y PMC9932951

[16] Fuertes E, Sunyer J, Gehring U et al Associations between air pollution and pediatric eczema, rhinoconjunctivitis and asthma: a meta-analysis of European birth cohorts. Environ Int 2020; 136:105474. 31962272 10.1016/j.envint.2020.105474

[17] Gabryszewski SJ, Dudley J, Grundmeier RW et al Early-life environmental exposures associate with individual and cumulative allergic morbidity. Pediatr Allergy Immunol 2021; 32:1089–93. 33616233 10.1111/pai.13486 PMC8249342

[18] Sigurdardottir ST, Jonasson K, Clausen M et al Prevalence and early-life risk factors of school-age allergic multimorbidity: the EuroPrevall-iFAAM birth cohort. Allergy 2021; 76:2855–65. 33934363 10.1111/all.14857 PMC8453757

[19] Stolk RP, Rosmalen JG, Postma DS et al Universal risk factors for multifactorial diseases LifeLines: a three-generation population-based study. Eur J Epidemiol 2008; 23:67–74. 18075776 10.1007/s10654-007-9204-4

[20] Zhang J, Loman L, Voorberg AN et al Prevalence of adult atopic dermatitis in the general population, with a focus on moderate-to-severe disease: results from the Lifelines Cohort Study. J Eur Acad Dermatol Venereol 2021; 35:e787–90. 34161629 10.1111/jdv.17471

[21] Zhang J, Loman L, Schuttelaar MLA. Limited health literacy and its associated health outcomes among adults with at least 2 atopic diseases. J Allergy Clin Immunol Pract 2023; 11:1429–38. 36634845 10.1016/j.jaip.2022.12.035

[22] ECRHS I Short Screening Questionnaire . Available at: https://www.ecrhs.org (last accessed 6 March 2026).

[23] Mahon GM, Koppelman GH, Vonk JM. Grandmaternal smoking, asthma and lung function in the offspring: the Lifelines cohort study. Thorax 2021; 76:441–7. 33542091 10.1136/thoraxjnl-2020-215232 PMC8070652

[24] Westerlaken-Van Ginkel CD, Vonk JM, Flokstra-de Blok BMJ et al Likely questionnaire-diagnosed food allergy in 78, 890 adults from the northern Netherlands. PLOS ONE 2020; 15:e0231818. 32401757 10.1371/journal.pone.0231818 PMC7219708

[25] van Erpecum C-PL, van Zon SKR, Bültmann U et al The association between fast-food outlet proximity and density and Body Mass Index: findings from 147,027 Lifelines Cohort Study participants. Prev Med 2022; 155:106915. 34922992 10.1016/j.ypmed.2021.106915

[26] Knol F, Boelhouwer J, Veldheer V. Statusontwikkeling van wijken in Nederland 1998–2010. Den Haag: Sociaal en Cultureel Planbureau (SCP), 2012.

[27] Brands MJ, Loman L, Schuttelaar MLA. Exposure and work-­related factors in subjects with hand eczema: data from a cross-sectional questionnaire within the Lifelines Cohort Study. Contact Dermatitis 2022; 86:493–506. 35122264 10.1111/cod.14066 PMC9314613

[28] Cohen J . Statistical Power Analysis for the Behavioral Sciences, 2nd edn. Hillsdale, NJ: Lawrence Erlbaum Associates, 1988.

[29] Moira APD, Pearce N, Pedersen M et al The influence of ­early-life animal exposure on the risk of childhood atopic dermatitis, asthma and allergic rhinoconjunctivitis: findings from the Danish National Birth Cohort. Int J Epidemiol 2023; 52:1231–42. 37018630 10.1093/ije/dyad040 PMC10396419

[30] Ojwang V, Nwaru BI, Takkinen H-M et al Early exposure to cats, dogs and farm animals and the risk of childhood asthma and allergy. Pediatr Allergy Immunol 2020; 31:265–72. 31829464 10.1111/pai.13186

[31] Lødrup Carlsen KC, Roll S, Carlsen K-H et al Does pet ownership in infancy lead to asthma or allergy at school age? Pooled analysis of individual participant data from 11 European birth cohorts. PLOS ONE 2012; 7:e43214. 22952649 10.1371/journal.pone.0043214 PMC3430634

[32] Standl M, Budu-Aggrey A, Johnston LJ et al Gene–environment interaction affects risk of atopic eczema: population and in vitro studies. Allergy 2025; 80:2201–12. 40462597 10.1111/all.16605 PMC12368900

[33] Schuttelaar MLA, Kerkhof M, Jonkman MF et al Filaggrin mutations in the onset of eczema, sensitization, asthma, hay fever and the interaction with cat exposure. Allergy 2009; 64:1758–65. 19839980 10.1111/j.1398-9995.2009.02080.x

[34] Nafstad P, Magnus P, Gaarder PI et al Exposure to pets and atopy-related diseases in the first 4 years of life. Allergy 2001; 56:307–12. 11284797 10.1034/j.1398-9995.2001.00881.x

[35] Svanes C, Heinrich J, Jarvis D et al Pet-keeping in childhood and adult asthma and hay fever: European community respiratory health survey. J Allergy Clin Immunol 2003; 112:289–300. 12897734 10.1067/mai.2003.1596

[36] Burney PG, Luczynska C, Chinn S et al The European community respiratory health survey. Eur Respir J 1994; 7:954–60. 8050554 10.1183/09031936.94.07050954

[37] Lu C, Liao H, Liu Z et al Association between early life exposure to indoor environmental factors and childhood asthma. Build Environ 2022; 226:109740.

[38] Apfelbacher C, Frew E, Xiang A et al Assessment of pet exposure by self-report in epidemiological studies of allergy and asthma: a systematic review. J Asthma 2016; 63:363–73. 10.3109/02770903.2015.1099161 26539692

[39] Zhong Z, Chen M, Dai S et al Association of cesarean section with asthma in children/adolescents: a systematic review and meta-analysis based on cohort studies. BMC Pediatr 2023; 23:571. 37974127 10.1186/s12887-023-04396-1 PMC10652517

[40] Pyrhönen K, Kulmala P. Delivery mode and the incidence of atopic sensitization and food allergy in a Finnish child population. Pediatr Allergy Immunol 2022; 33:e13584. 34184325 10.1111/pai.13584

[41] Gerlich J, Benecke N, Peters-Weist AS et al Pregnancy and peri­natal conditions and atopic disease prevalence in childhood and adulthood. Allergy 2018; 73:1064–74. 29193127 10.1111/all.13372

[42] Liu X, Zhou J, Chen J et al Risk of asthma and allergies in children delivered by cesarean section: a comprehensive systematic review. J Allergy Clin Immunol Pract 2024; 12:2764–73. 38908434 10.1016/j.jaip.2024.06.022

[43] Mubanga M, Lundholm C, Rohlin ES et al Mode of delivery and offspring atopic dermatitis in a Swedish nationwide study. Pediatr Allergy Immunol 2023; 34:e13904. 36705040 10.1111/pai.13904 PMC10107099

[44] Lehtimäki J, Thorsen J, Rasmussen MA et al Urbanized microbiota in infants, immune constitution, and later risk of atopic diseases. J Allergy Clin Immunol 2021; 148:234–43. 33338536 10.1016/j.jaci.2020.12.621

[45] Jarvis D, Chinn S, Luczynska C et al The association of family size with atopy and atopic disease. Clin Exp Allergy 1997; 27:240–5. 9088649

[46] Svanes C, Jarvis D, Chinn S et al Childhood environment and adult atopy: results from the European Community Respiratory Health Survey. J Allergy Clin Immunol 1999; 103:415–20. 10069874 10.1016/s0091-6749(99)70465-3

[47] Filipiak B, Heinrich J, Schäfer T et al Farming, rural lifestyle and atopy in adults from southern Germany – results from the MONICA/KORA study Augsburg. Clin Exp Allergy 2001; 31:1829–38. 11737033 10.1046/j.1365-2222.2001.01246.x

[48] Tian J, Zhang D, Yang Y et al Global epidemiology of atopic dermatitis: a comprehensive systematic analysis and modelling study. Br J Dermatol 2023; 190:55–61. 37705227 10.1093/bjd/ljad339

[49] Yim Y, Jo H, Park S et al Sex-specific and long-term trends of asthma, allergic rhinitis, and atopic dermatitis in South Korea, 2007–2022: a nationwide representative study. Int Arch Allergy Immunol 2025; 186:166–83. 39284302 10.1159/000540928 PMC11793097

[50] Keller T, Hohmann C, Standl M et al The sex-shift in single disease and multimorbid asthma and rhinitis during puberty – a study by MeDALL. Allergy 2018; 73:602–14. 28960325 10.1111/all.13312 PMC5836860

[51] Hohmann C, Keller T, Gehring U et al Sex-specific incidence of asthma, rhinitis and respiratory multimorbidity before and after puberty onset: individual participant meta-analysis of five birth cohorts collaborating in MeDALL. BMJ Open Respir Res 2019; 6:e000460. 10.1136/bmjresp-2019-000460 PMC6797252 31673365

[52] Gutiérrez-Brito JA, Lomelí-Nieto JÁ, Muñoz-Valle JF et al Sex hormones and allergies: exploring the gender differences in immune responses. Front Allergy 2025; 5:1483919. 39840271 10.3389/falgy.2024.1483919 PMC11747284

[53] Laffont S, Guéry J-C. Deconstructing the sex bias in allergy and autoimmunity: from sex hormones and beyond. Adv Immunol 2019; 142:35–64. 31296302 10.1016/bs.ai.2019.04.001

[54] Klijs B, Scholtens S, Mandemakers JJ et al Representativeness of the LifeLines Cohort Study. PLOS ONE 2015; 10:e0137203. 26333164 10.1371/journal.pone.0137203 PMC4557968

[55] FEDIAF . Annual report 2025. Available at: https://europeanpetfood.org/wp-content/uploads/2025/12/FEDIAF-AR-2025_Online.pdf (last accessed 6 March 2026).

