# Supplementary material for: Association of atopic multimorbidity with childhood pet exposure and caesarean section delivery: a retrospective study from the Lifelines Cohort Study
Source: Skin Health Dis. 2026 Jun 9;6(4):557–66. doi: 10.1093/skinhd/vzag070 (PMC13425007; doi:10.1093/skinhd/vzag070)
Supplement: vzag070_Supplementary_Data [file vzag070_supplementary_data.zip › Supporting_Information.docx]

**Supporting Information**

**Figure S1.** **Venn diagram of overlapping atopic diseases among participants with complete information**

AD: atopic dermatitis; FA: food allergy; AR: allergic rhinitis.

Note: Among participants with complete information on all four diseases (N = 44,559), those who did not have any of the atopic diseases were not included in this figure.

**Figure S2. Age-group–specific adjusted odds ratios from models including exposure × age-group interaction terms**

Note: This forest plot displays age-group–specific adjusted odds ratios (ORs) with 95% confidence intervals (CIs) and P values for each environmental exposure from models including exposure × age-group interaction terms. Some ORs and corresponding 95% CIs for the ≥66 years age group are not shown because the model produced infinite estimates.

**Table S1. Description of included environmental exposures**

| **Exposures** | **Questions in Lifelines** | **Answer options in Lifelines** | **Categories in the current study** |
| --- | --- | --- | --- |
| Birth weight | What was your birth weight? | Written answers | [0] Normal (2.5-4.0 kg, ref.)  [1] Low (<2.5 kg)  [2] High (>4.0 kg)  [*Missing*] If written answers > mean + 3*SD; or written answers < mean - 3*SD |
| Gestational age | What was the pregnancy duration when you were born (normally the gestational periods is 40 weeks)? | Written answers | [0] Full term (39-40 weeks, ref.)  [1] Preterm (<37 weeks)  [2] Early term (37-38 weeks)  [3] Late/post-term (≥41 weeks) |
| Delivery mode | How were you born? | 1. Normal vaginal delivery  2. Assist birth (vaginal delivery with use of forceps or vacuum pump)  3. Caesarean section  4. I don’t know | [0] Vaginal delivery (1-2, ref.)  [1] Caesarean section (3)  [*Missing*] (4) |
| Breastfeeding | How long were you breastfed as a baby? | 1. I was not breastfed  2. I was breastfed but I don’t know for how long  3. 0-2 weeks  4. 2-4 weeks  5. 1-3 months  6. 3-6 months  7. 6-12 months  8. For more than 1 year  9. I don’t know whether I was breastfed | [0] Never (1, ref.)  [1] Yes (2-8)  [*Missing*] (9) |
| Prenatal maternal smoking | Did your mother smoke when she was pregnant with you? | 1. No, my mother never smoked  2. No, my mother started smoking after the pregnancy  3. No, my mother stopped smoking before the pregnancy  4. Yes, but my mother has stopped smoking or has smoked less during the pregnancy  5. Yes, my mother has smoked as usual during the pregnancy  6. I don’t know | [0] No (0-3, ref.)  [1] Yes (4-5)  [*Missing*] (6) |
| Smoke exposure in childhood | 1a. Has your father ever smoked regularly during your childhood?  1b. Has your mother ever smoked regularly during your childhood? | Shared options for questions 1a, 1b:  0. No  1. Yes | [0] No (all “no” for 1a and 1b, ref.)  [1] Yes (any “yes” in 1a/1b) |
| Pet ownership ever | 2a. Was there a cat in your home during your first year of life?  2b. Was there a dog in your home during your first year of life?  2c. Was there a cat in your home when you were 1-4 years old?  2d. Was there a dog in your home when you were 1-4 years old?  2e. Was there a cat in your home when you were 5-15 years old?  2f. Was there a dog in your home when you were 5-15 years old? | Shared options for questions 2a to 2f:  0. No  1. Yes | [0] No pet (all “No” from 2a to 2f, ref.)  [1] Yes (any “Yes” from 2a to 2f) |
| Type of pet |  |  | [0] No pet (all “No” from 2a to 2f, ref.)  [1] Dog only (any “Yes” in 2b/2d/2f AND (all “No” in 2a/2c/2e)  [2] Cat only (any “Yes” in 2a/2c/2e AND (all “No” in 2b/2d/2f)  [3] Both dog and cat (If any “Yes” in 2a/2c/2e AND any “Yes” in 2b/2d/2f) |
| Age of having pet |  |  | [0] During the first year (any “Yes” in 2a/2b, ref.)  [1] After 1 years old (all “No” in 2a/2b AND any “Yes” from 2c to 2f) |
| Farm-living | What is the best description of the place where you lived most of the time when you were younger than 5 years old? | 1. Farm  2. Rural village  3. Small city/ large village  4. Suburb of a large city  5. City center  6. Other situation | [0] Non-farm (2-5, ref.)  [1] Farm (1)  [*Missing*] (6) |
| Urbanity | By linking Lifelines participants’ municipal codes to the Statistics Netherlands (CBS) code, to obtain the information on urbanity, which is based on environmental addresses density | 1. Rural (<500 addresses/km^2^)  2. Semi-rural (500-1000 addresses/km^2^)  3. Intermediate urban-rural (1000-1500 addresses/km^2^)  4. Semi-urban (1500-2500 addresses/km^2^)  5. Urban (≥2500 addresses/km^2^) | [0] Urban (3-5, ref.)  [1] Rural (1-2) |

SD: standard deviation; ref.: reference group.

**Table S2. Non-responder analysis comparing the study population with the non-study population**

|  | **Non-study population (N=99,037)** | **Study population**  **(N=28,791)** | ***P* value** | **Effect size (95% CI)** |
| --- | --- | --- | --- | --- |
| **Age, yrs.** mean ± SD | 51.90 ± 12.98 | 56.88 ± 11.90 | **<0.001** | -0.39 (-0.40, -0.38) |
| **Sex** (N, %) |  |  | **<0.001** | 0.02 (0.00, 0.03) |
| - Male | 41819 (42.2) | 11918 (41.4) |  |  |
| - Female | 57218 (57.8) | 16873 (58.6) |  |  |
| **Birth weight** (N, %), kg |  |  | **0.020** | 0.01 (0.00, 0.01) |
| - Low, <2.5 | 5240 (8.2) | 1443 (7.7) |  |  |
| - Normal, 2.5-4 | 51418 (80.0) | 15184 (80.8) |  |  |
| - High, >4 | 7594 (11.8) | 2168 (11.5) |  |  |
| *Missing* | *34785* | *9996* |  |  |
| **Gestational age** (N, %), weeks |  |  | **<0.001** | 0.04 (0.03, 0.05) |
| - Preterm, <37 | 3535 (5.2) | 919 (4.6) |  |  |
| - Early term, 37-38 | 6080 (9.0) | 1580 (7.9) |  |  |
| - Full term, 39-40 | 44848 (66.4) | 14130 (70.9) |  |  |
| - Late/post-term, ≥41 | 13093 (19.4) | 3290 (16.5) |  |  |
| *Missing* | *31481* | *8872* |  |  |
| **Delivery mode** (N, %) |  |  | **<0.001** | -0.07 (-0.08, -0.05) |
| - Vaginal delivery | 92048 (97.0) | 27210 (98.0) |  |  |
| - Caesarean section | 2889 (3.0) | 553 (2.0) |  |  |
| *Missing* | *4100* | *1028* |  |  |
| **Breastfeeding ever** (N, %) |  |  | **<0.001** | 0.08 (0.06, 0.10) |
| - No | 22170 (38.1) | 5501 (34.2) |  |  |
| - Yes | 36094 (61.9) | 10561 (65.8) |  |  |
| *Missing* | *40773* | *12729* |  |  |
| **Prenatal maternal smoking** (N, %) |  |  | **<0.001** | -0.12 (-0.14, -0.11) |
| - No | 69343 (82.2) | 21515 (86.6) |  |  |
| - Yes | 15064 (17.8) | 3323 (13.4) |  |  |
| *Missing* | *14630* | *3953* |  |  |
| **Smoke exposure in childhood** (N, %) |  |  | **<0.001** | 0.11 (0.10, 0.12) |
| - No | 24063 (28.5) | 5888 (23.7) |  |  |
| - Yes | 60344 (71.5) | 18950 (76.3) |  |  |
| *Missing* | *14630* | *3953* |  |  |
| **Pet ownership ever** (N, %) |  |  | **<0.001** | -0.06 (-0.07, -0.05) |
| - No | 25724 (26.0) | 8274 (28.8) |  |  |
| - Yes | 73167 (74.0) | 20495 (71.2) |  |  |
| *Missing* | *146* | *22* |  |  |
| **Type of pet** (N, %) |  |  | **<0.001** | 0.02 (0.01, 0.03) |
| - Dog only | 23694 (32.4) | 6343 (30.9) |  |  |
| - Cat only | 18900 (25.8) | 5742 (28.0) |  |  |
| - Both dog and cat | 30573 (41.8) | 8410 (41.0) |  |  |
| *Missing* | *25870* | *8296* |  |  |
| **Age of having pet** (N, %) |  |  | **<0.001** | 0.06 (0.05, 0.08) |
| - During the first year | 37212 (50.9) | 9785 (47.7) |  |  |
| - After 1 years old | 35955 (49.1) | 10710 (52.3) |  |  |
| *Missing* | *25870* | *8296* |  |  |
| **Farm living before 5 yrs.** (N, %) |  |  | **<0.001** | 0.12 (0.11, 0.13) |
| - Non-farm | 84474 (87.8) | 23451 (83.6) |  |  |
| - Farm | 11801 (12.2) | 4608 (16.4) |  |  |
| *Missing* | *2762* | *732* |  |  |
| **Urbanity** (N, %), addresses per km^2^ |  |  | **0.003** | 0.02 (0.01, 0.04) |
| - Urban (≥1000) | 31229 (31.5) | 8766 (30.4) |  |  |
| - Rural (<1000) | 67774 (68.5) | 20025 (69.6) |  |  |
| *Missing* | 34 | 0 |  |  |
