# Supplementary figures and images for: Association of atopic multimorbidity with childhood pet exposure and caesarean section delivery: a retrospective study from the Lifelines Cohort Study
Source: Skin Health Dis. 2026 Jun 9;6(4):557–66. doi: 10.1093/skinhd/vzag070 (PMC13425007; doi:10.1093/skinhd/vzag070)

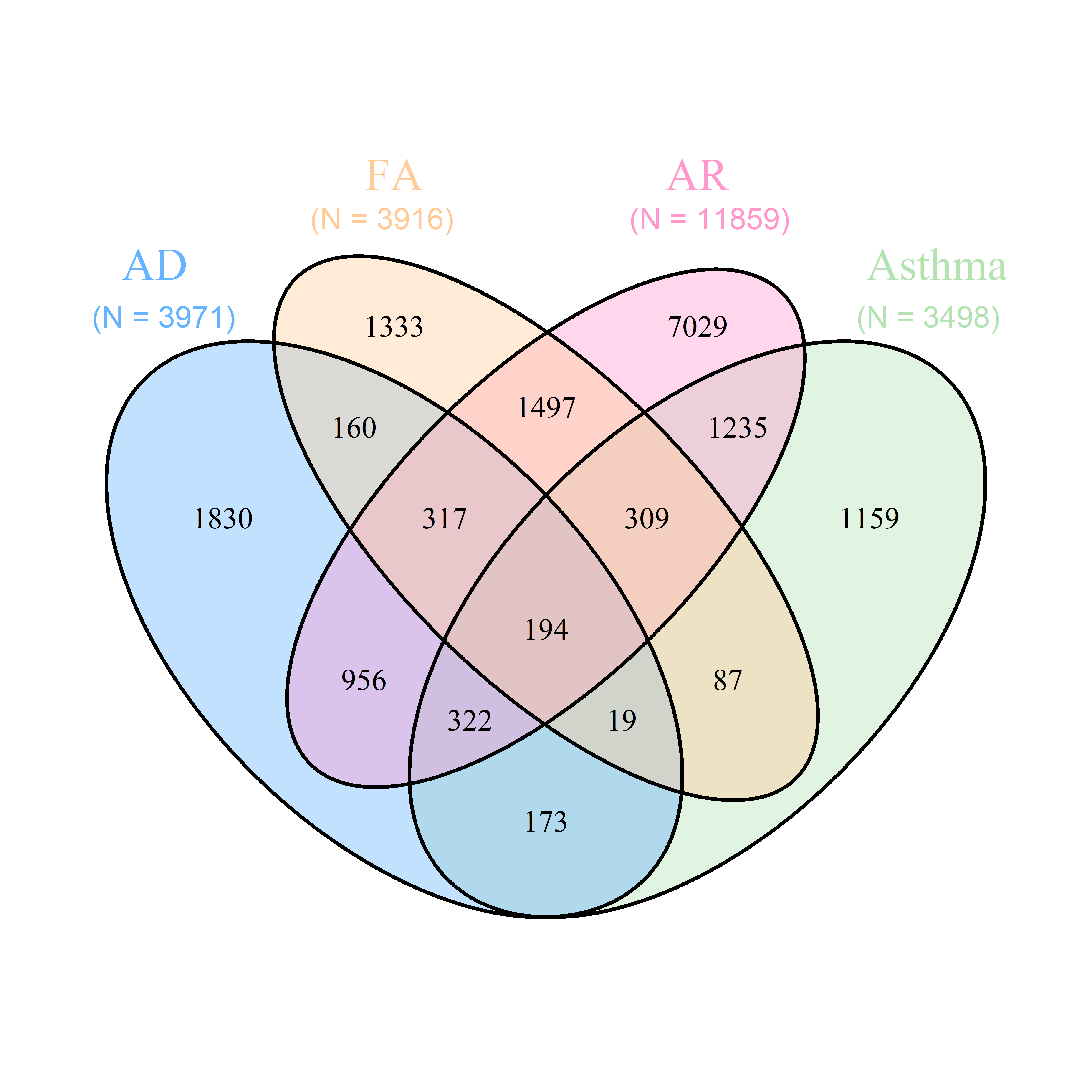

Supplement: vzag070_Supplementary_Data [file vzag070_supplementary_data.zip › Figure_S1.tiff]

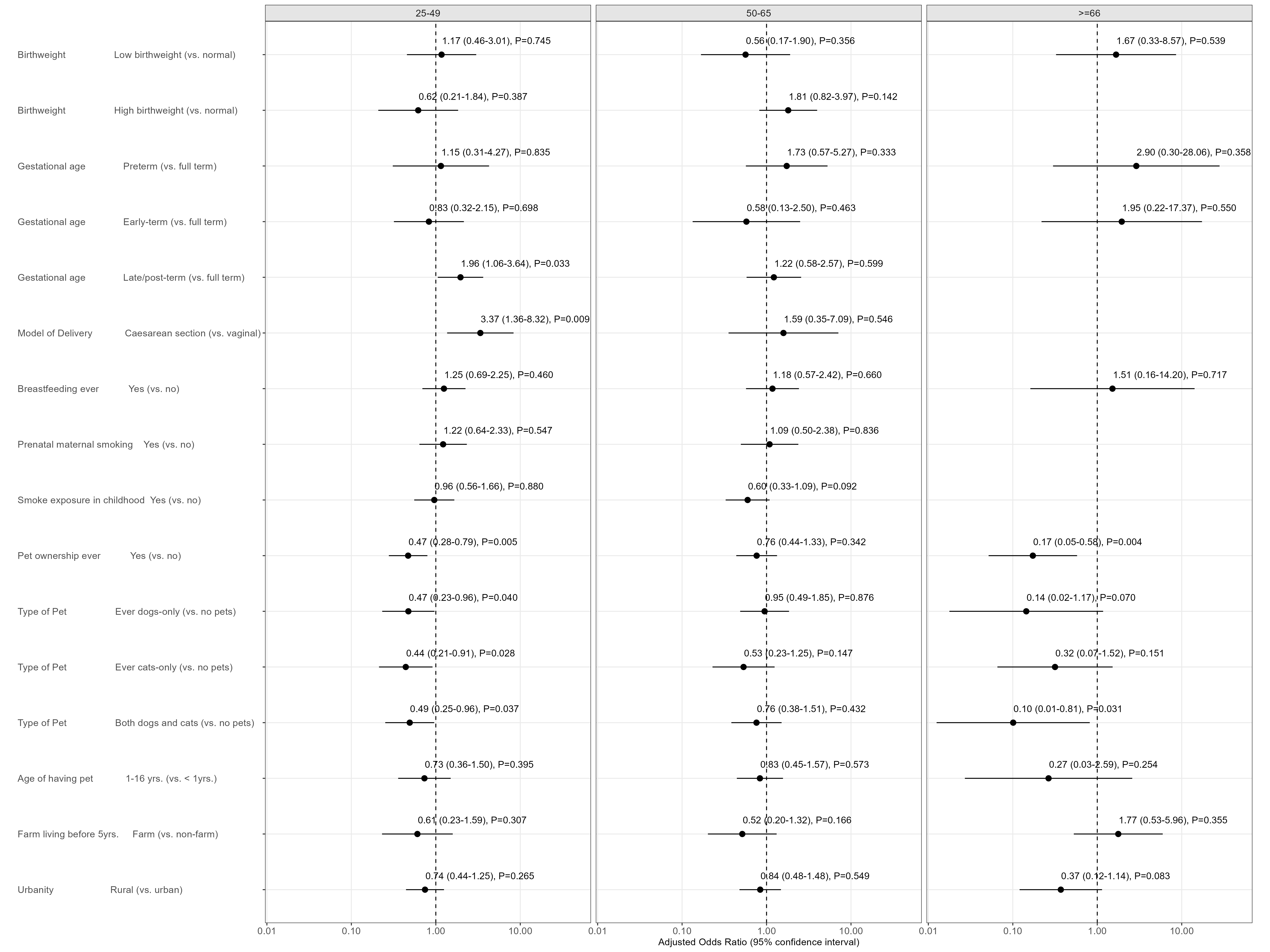

Supplement: vzag070_Supplementary_Data [file vzag070_supplementary_data.zip › Figure_S2_300dpi_lzw.tiff]
